# Uridine as a hub in cancer metabolism and RNA biology

**DOI:** 10.1038/s12276-025-01402-7

**Published:** 2025-08-14

**Authors:** Kyoung-Min Choi, Brennon A. Berard, Je-Hyun Yoon, Dohoon Kim

**Affiliations:** 1 Department of Oncology Science, University of Oklahoma, Oklahoma City, OK USA; 2 Department of Molecular, Cell and Cancer Biology, University of Massachusetts Chan Medical School, Worcester, MA USA; 3 Department of Pathology, University of Oklahoma, Oklahoma City, OK USA

**Keywords:** RNA decay, Cancer metabolism, Cancer therapeutic resistance

## Abstract

Uridine is the ubiquitous nucleoside form of the RNA base uracil. It occupies a prominent ‘hub’ position in energy metabolism; for example, it is metabolically linked to de novo pyrimidine biosynthesis and glycolysis and biologically linked to diverse processes, such as RNA synthesis/degradation and glycosylation. It is a vital interorgan ‘currency’ nutrient readily imported by mammalian cells, and its supplementation can exert both cytoprotective and toxic effects, for which the underlying mechanisms are poorly understood. Importantly, it is a route by which the decay of RNA can be repurposed as an alternative fuel source under nutrient-limiting conditions to aid in tumor initiation, development and metastasis. Here we explain how the upstream inputs and downstream metabolic fates of uridine influence cancer traits and illustrate both established and hypothetical strategies targeting uridine metabolism for cancer therapy.

## Uridine metabolism links energy and cancer progression

Uridine is a crucial molecule in our bodies, involved in many biological processes. This study explores how cancer cells use uridine and how its biosynthetic pathway might be targeted for cancer therapy. Uridine circulates in the blood and is produced mainly in the liver. Cells take up uridine and convert it into other molecules, such as uridine monophosphate, that are essential for cell growth and survival. The study highlights that uridine can support cancer cell growth, especially when glucose is scarce, by providing an alternative energy source. Various methods have been used to study the roles of uridine in cancer, including examining how it is metabolized and how it affects cancer cell metabolism such as glycolysis, and found that disrupting uridine metabolism could potentially hinder cancer cell growth.

This summary was initially drafted using artificial intelligence, then revised and fact-checked by the author.

## Overview of uridine

**Fig. 1:**
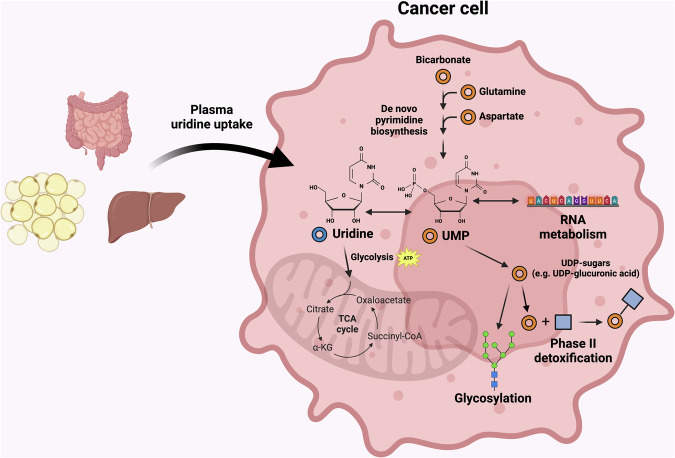
Overview of uridine metabolism in a cancer cell. Uridine can be directly taken up from plasma, produced via the pyrimidine biosynthesis pathway, or obtained from RNA decay. It can then contribute to RNA/DNA synthesis, the TCA cycle, glycosylation and glucuronidation. Figure created using Biorender (https://biorender.com/).

Uridine is a nucleotide that is common to all life forms and occupies a unique and complex position in metabolism from both an intracellular and intercellular perspective. In the intracellular metabolic network, it is adjacent to uridine monophosphate (UMP) and thus it can either be formed via the de novo pyrimidine biosynthetic pathway or, conversely, provide UMP in cells lacking it (Part 2). Thus, uridine metabolism is intertwined with key biological processes such as DNA/RNA synthesis, particularly RNA synthesis, as it uses the uridine base, as well as glycosylation, which utilizes uridine diphosphate (UDP) as the sugar carrier molecule (Fig. 1). Furthermore, its catabolism can support both glycolysis and the tricarboxylic acid (TCA) cycle (Part 3).

From an intercellular/organismal perspective, uridine is also an important interorgan nutrient. Uridine, the natural nucleoside precursor of UMP, is reported to exist in human plasma at 3–8 μM, which is substantially greater than other nucleosides^1–3^, and is predominantly produced by the liver or adipose tissue for utilization across other tissues^4^. In addition, in human serum, UMP can be rapidly dephosphorylated to uridine in the presence of 5′-nucleotidase^5,6^. Thus, cells can readily obtain uridine from the environment, and the administration of uridine is cytoprotective in a wide range of contexts, including chemotherapy and ischemia^7–9^. On the other hand, the disruption of uridine homeostasis by the knockout of uridine phosphorylase can cause spontaneous tumors in mice and uridine treatment of cells can cause DNA damage and toxicity, suggesting that uridine homeostasis is essential^10,11^.

All of these aspects have implications for cancer cells and here we review the upstream metabolic factors that lead to uridine formation and the downstream impact of uridine metabolism from the perspective of cancer cells. We discuss how cancer cells obtain uridine, what they use uridine for and how any of these steps may be targeted for cancer therapy.

## Part 1: uridine is a metabolic ‘currency’

While most metabolites, such as nicotinamide adenine nucleotide hydrogen and glycolytic intermediates, are produced and metabolized intracellularly, some such as glucose and ketone bodies (acetoacetate, 3-β-hydroxybutyrate and acetone), represent organism-level metabolic ‘currencies’—metabolites that are regulated at the whole-organism level, widely circulated and provided through the plasma to a variety of cell types^12–15^. For example, multiple organs in the body utilize glucose and regulate its circulatory levels^14,16^. In the same manner, uridine in the plasma is freely available for uptake by cells and uridine abundance is tightly regulated through various mechanisms, particularly via synthesis and degradation in the liver^17,18^ (Fig. 1). Under normal-fed states, the liver is the primary source of uridine biosynthesis as well as catabolism in the body^4^. However, under fasted states, pyrimidine biosynthesis from adipose tissue results in compensatory overproduction of uridine^4^. In addition to fasting, various physiological factors, such as alcohol consumption, diet and exercise, strongly regulate plasma uridine levels^17,19,20^. For example, uridine levels can be elevated with the consumption of certain uridine-rich foods, such as beer, or via ethanol itself^19,21^.

**Fig. 2:**
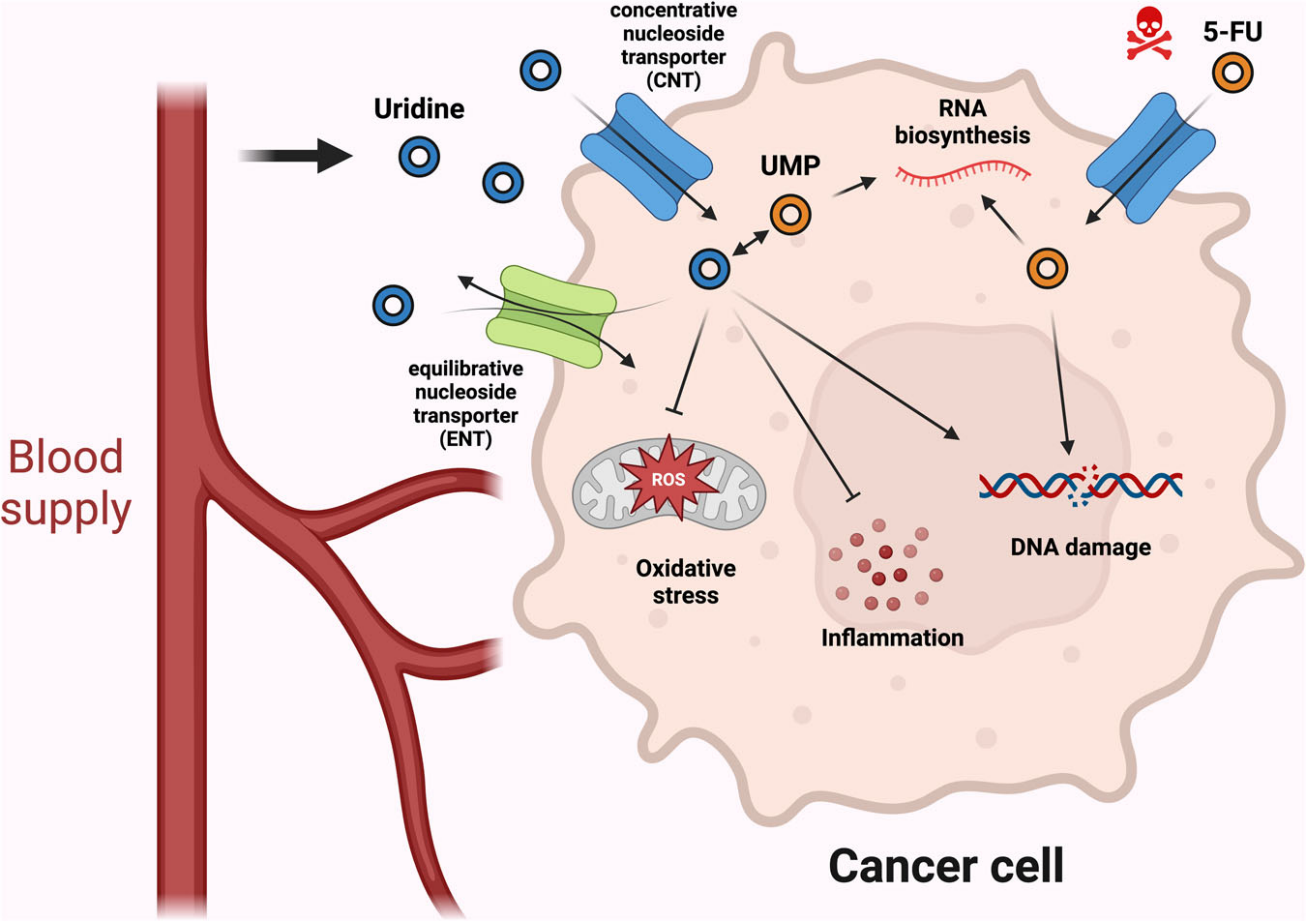
Summary of known consequences of uridine administration in cancer. Uridine can be imported into cancer cells via CNTs and ENTs to competitively inhibit the incorporation of 5-FU into RNA, minimizing gastrointestinal toxicity while retaining its cytotoxicity to cancer cells. Uridine also has various cytoprotective properties and suppresses oxidative stress and inflammation. ROS, reactive oxygen species. Figure created using Biorender (https://biorender.com/).

Uridine, in turn, is readily taken up by cells, as demonstrated in an organism context and in cell/tissue culture systems. Uridine uptake is mediated by both equilibrative nucleoside transporters (ENTs) and concentrative nucleoside transporters (CNTs), which can deliver a range of nucleosides (ENTs and CNTs)^22^ (Fig. 2), and the injection of supraphysiologic uridine can result in rapid uptake in most tissues, especially renal tissues^23^. Uridine uptake in various cell lines is evident^24,25^, and radiolabeled uridine uptake and incorporation into RNA has even been used as a measure of cell viability^26^.

### Uridine uptake in cancer

Intriguingly, in stark contrast to glucose, whose uptake and downstream utilization have been central points of focus in cancer metabolism research^27,28^, relatively little is known about the characteristics of uridine uptake and utilization by cancer cells. In particular, there is no consensus on whether certain cancers have elevated uridine uptake and for what purpose; however, much on uridine uptake can be extrapolated from studies on nucleoside analog uptake in tumors, as nucleoside transporters are also expected to mediate uridine uptake. In fact, labeled uridine uptake is often used as a proxy for the nucleoside analog uptake capacity of tumors^29,30^. Similarly, the overexpression of specific nucleoside transporters has been linked with tumor sensitivity to nucleoside analogs, and the loss of transporter expression has been linked with resistance^31–35^. However, these studies did not directly compare the uridine uptake of tumor tissues with that of normal tissues; thus, it remains an open question whether certain subtypes of tumors may have increased uridine uptake and be particularly dependent on uridine as a metabolic currency.

### Consequences of uridine uptake

Uridine exerts various activities across organs, supporting its role as a currency metabolite. For example, its administration regulates reproductive organ function, vasoregulation and neuronal activity^36–38^, demonstrating that in normal physiology, uridine provided from the plasma is indeed of consequence to various cells. In one of the best-documented and cancer-related applications, uridine has been used in forms such as uridine triacetate as a cotreatment to reduce patient toxicity during nucleoside chemotherapy, such as with 5-fluorouracil (5-FU), while maintaining antitumor toxicity^7,39^. It is postulated that the protective effect occurs by competitively limiting the uptake of 5-FU into RNA, which is a major source of gastrointestinal toxicity^40^ (Fig. 2). Uridine has even been identified as a potent inducer of tissue regeneration^41^. Uridine supplementation has been successfully implemented for rare disorders such as orotic aciduria and carbamoyl phosphate synthetase II, aspartate transcarbamylase and dihydroorotase (CAD) deficiency, which are disorders in which enzymes responsible for pyrimidine biosynthesis, such as uridine monophosphate synthetase (UMPS) or CAD, are mutated; thus, uridine provides alternative precursors for pyrimidine synthesis^42,43^.

Intriguingly, uridine has various effects on cells in tissue culture. For example, it is cytoprotective in various conditions of stress, such as rescuing viability against oxidative phosphorylation or mitochondrial deficiency^44,45^, glucose deprivation^8^, inflammation and oxidative stress^9^. In oxidative phosphorylation or mitochondrial deficiency, uridine supplementation provides alternative precursors for pyrimidine biosynthesis, which are needed as the pyrimidine biosynthetic enzyme dihydroorotate dehydrogenase (DHODH) is dependent on the electron transport chain^46^. However, there may be additional undiscovered mechanisms of uridine-based cytoprotection, such as during oxidative stress. On the other hand, high levels of uridine can negatively affect cell viability, such as by inducing ferroptosis in hepatocellular carcinoma cells^11^ and causing DNA damage and p53 activation in a process referred to as ‘uracil damage’^10^. The same study revealed that the loss of uracil processing via uridine phosphorylase, resulting in uridine hyperaccumulation, can be carcinogenic. However, the aforementioned successful use of uridine supplementation in protecting against chemotherapy-induced toxicity or in-born metabolic disorders, without any noted side effects^42,43^, suggests that, in moderation, uridine can be safe and beneficial.

Thus, it is clear that uridine is a currency metabolite with a wide range of consequences for the cells that access it. To understand the underlying mechanisms of the effects of uridine in cancer cells and explore future options for therapeutic exploitation, it is important to review what is known about the downstream metabolic fates of uridine metabolism, as outlined below. Additionally, we consider other sources of uridine aside from the extracellular environment, particularly the de novo pyrimidine biosynthesis pathway, where the pathway end-product UMP is interconverted with uridine. Additionally, we consider nucleic acids themselves, particularly RNA, as sources of uridine upon their decay. In the sections below, we explore the various connections of uridine with other metabolic pathways, the potential importance of uridine from the perspective of cancer cells and how these connections may be potential points of vulnerability.

## Part 2: upstream contributors to uridine metabolism

### UMP as a source of uridine and vice versa

The metabolic fates of uridine and UMP are intertwined and the two molecules are only a bidirectional metabolic step removed from each other. Uridine, obtained from plasma, can be converted to UMP via uridine cytidine kinases (UCKs). On the other hand, UMP can be biosynthesized via a pathway that utilizes glutamine and bicarbonate to produce carbamoyl phosphate, followed by the incorporation of aspartate to generate carbamoyl aspartate as the initial step^47,48^. It can also be obtained from the breakdown of RNA and then converted to uridine via 5′ nucleotidases^24^. As described in the following sections, the relationship is bidirectional and fuels various requirements of cancer cells, including glycolysis occurring downstream of uridine or RNA synthesis and glycosylation occurring downstream of UMP.

### Uridine to UMP via UCKs

**Fig. 3:**
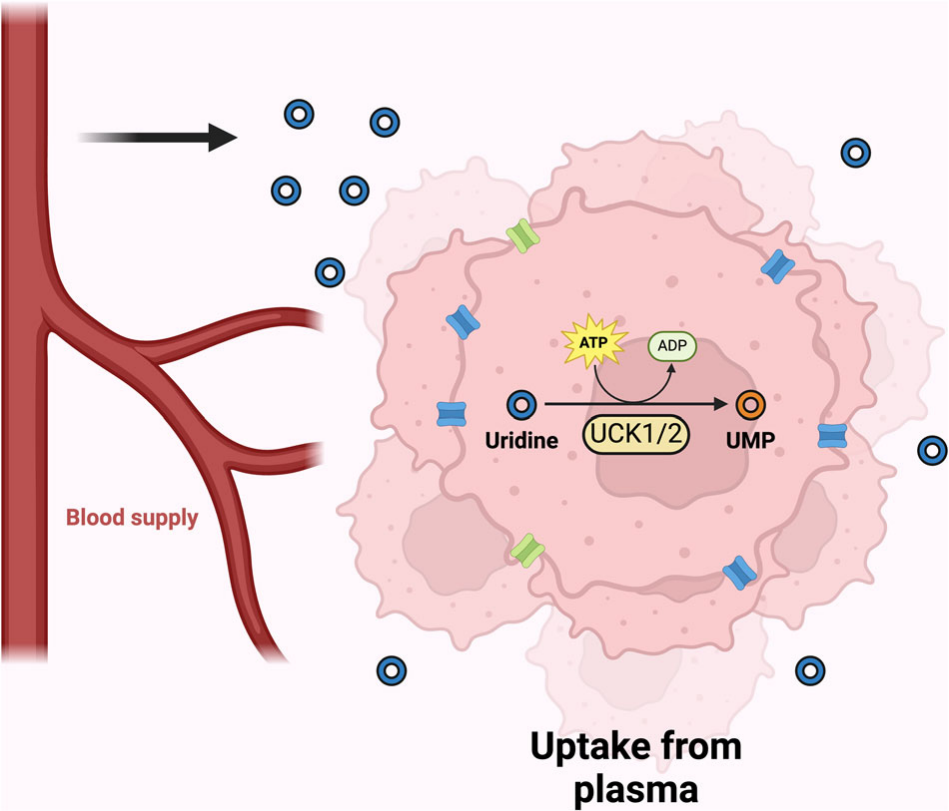
Uridine taken up from plasma can be converted to UMP via UCKs. Figure created using Biorender (https://biorender.com/).

Uridine can be phosphorylated to UMP via UCK, UCK1 and UCK2 in humans^49,50^ (Fig. 3). UCK1 is present in both cancer and normal cells, whereas UCK2 is preferentially expressed in various cancer cells, including neuroblastoma cells^51–53^. Given that uridine obtained extracellularly can contribute to UMP processes, radiolabeled uridine is readily incorporated into RNA^26^ and uridine administration induces glycosylation in cells and in vivo^36,54^.

UCK enzymes have been explored in cancer therapy, mainly for their role in the metabolic activation of ribonucleoside analog chemotherapeutics. UCK phosphorylates the ribonucleoside analog TAS 106 (3′-ethynylcytidine) to generate 3′-ethynylcytidine-5′-monophosphate^51^ that undergoes two phosphorylation steps to be converted into 3′-ethynylcytidine-5′-triphosphate, which inhibits RNA polymerases and exerts an antitumor effect^51^. TAS 106 showed excellent antitumoral activity in HT-1080 (half-maximum inhibitory concentration (IC_50_) of 0.01 μM) and NUGC-3 (IC_50_ of 0.35 μM) cells^51^. Notably, the level of UCK2, rather than that of UCK1, is closely correlated with the sensitivity of various cancer cells, including pancreatic cancer cells, to TAS 106 (ref. ^55^). The cytidine analog fluorocyclopentenylcytosine, RX-3117, is activated by UCK2, and its di- and triphosphorylated metabolites are incorporated into RNA and DNA, inhibiting gene synthesis^56–58^. RX-3117 exhibited potent antitumor activity in MDA-MB-231 (IC_50_ of 0.18 μM), NCIH226 (IC_50_ of 0.25 μM) and HCT116 (IC_50_ of 0.19 μM) cells^59,60^. Notably, UCK1 knockdown by short interfering RNA did not affect the sensitivity of A549 and SW1573 cells to RX-3117, whereas UCK2 knockdown reduced the sensitivity of A549 and SW1573 cells to RX-3117 (ref. ^61^). While such roles of UCK enzymes are well known, less is known about the implications of blocking the uridine-to-UMP flux that would occur upon UCK disruption, which is a potential avenue for therapy.

In addition to its catalytic role as a UCK, UCK2 has recently been shown to play important catalysis-independent roles, particularly in activating oncogenic signaling pathways such as the EGFR‒Akt axis through direct binding^62,63^. Thus, in addition to catalytic inhibitors, strategies that target UCK2 via degradation constitute an intriguing therapeutic avenue.

### Glutamine to UMP via the pyrimidine biosynthesis pathway

**Fig. 4:**
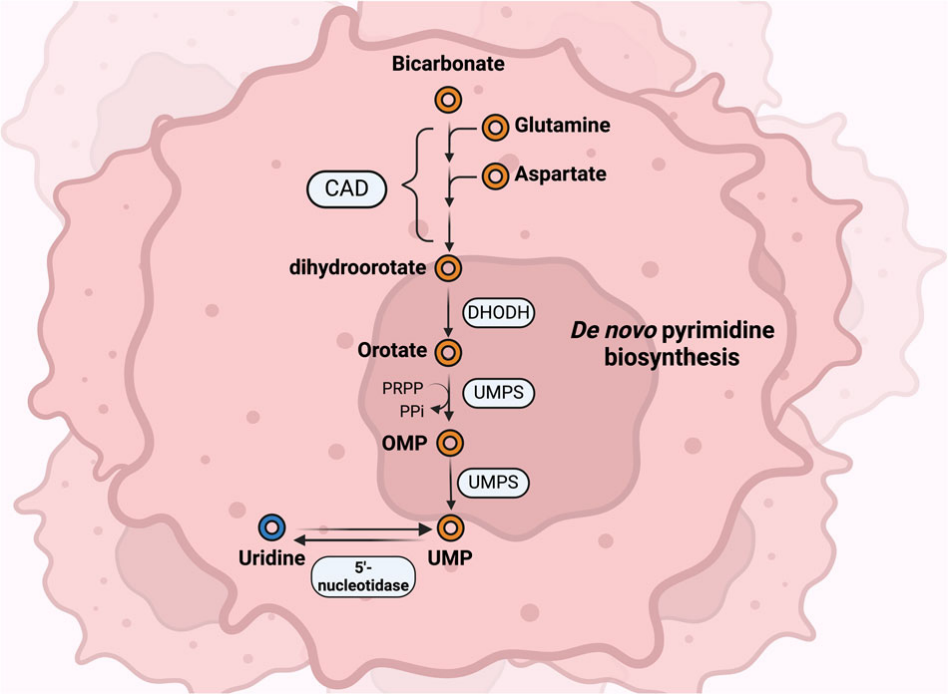
De novo pyrimidine biosynthesis pathway as a source of uridine. The UMP produced by this pathway can be converted to uridine via 5′-nucleotidases. Figure created using Biorender (https://biorender.com/).

The initial three reactions of the UMP de novo synthesis pathway, also known as the pyrimidine biosynthesis pathway, are catalyzed by CAD, generating dihydroorotate from bicarbonate, glutamine and aspartate^64,65^ (Fig. 4). Dihydroorotate is processed to orotate by DHODH on the outer face of the inner mitochondrial membrane, and orotate is metabolized in two steps, which are both catalyzed by the bifunctional enzyme UMPS^66,67^. The N-terminal domain of UMPS first catalyzes the conversion of orotate to orotidine monophosphate (OMP), utilizing phosphoribosyl diphosphate (PRPP) as a cosubstrate. The C-terminal domain of UMPS then catalyzes the conversion of OMP into UMP. Interestingly, the activity of this pathway is low in nonproliferating (that is, most normal) cells, whose demand for pyrimidines is largely met by salvage pathways^68^. On the other hand, this pathway is generally highly active in proliferating cells, which have high pyrimidine demand^69^. This finding raises the possibility that UMP produced in cancer cells can feed into uridine production. Conversely, owing to the high pyrimidine demand for RNA/DNA synthesis, uridine uptake by cancer cells may feed into the formation of UMP and subsequent formation of pyrimidine, which is used to fuel cancer growth. Future research, such as using labeled glutamine as a precursor for UMP formation, is needed to elucidate the exact relationship between uridine uptake and the pyrimidine biosynthesis pathway in cancer cells.

Among the enzymes involved in de novo synthesis pathways, the expression and activity of DHODH are reported to be directly associated with cancer progression^70^. Arava (leflunomide), an inhibitor of DHODH, received United States Food and Drug Administration approval in 1998^71^, and its active metabolite aubagio (teriflunomide) received Food and Drug Administration approval in 2012^71^. DHODH, in addition to its role in pyrimidine biosynthesis, has received attention for its role in suppressing ferroptosis^72^. However, the observed effects of the DHODH inhibitor brequinar on sensitization to ferroptosis may be due to the inhibition of another enzyme, FSP1 (ref. ^73^); thus, further efforts are warranted to clarify the roles of DHODH in cancer cells.

### Cytidine salvage to uridine

Uridine can be formed through the transformation of cytidine salvaged from the bloodstream^74^. This process is dependent on cytidine deaminase (CDA), which converts cytidine to uridine. CDA has been reported to be resistant to cytidine analogs, including cytosine arabinoside and 2′,2′-difluorodeoxycytidine (gemcitabine), since CDA metabolizes cytidine analogs into inactive metabolites^75,76^. CDA inhibitors have been developed, but primarily explored for their ability to negate the resistance-promoting property of CDA^75,76^. Whether CDA is relevant to cancer cells as a significant source of uracil, relative to other sources described, remains an interesting research direction and potential application for CDA inhibitors.

## Part 3: RNA decay as an intracellular source of uridine

**Fig. 5:**
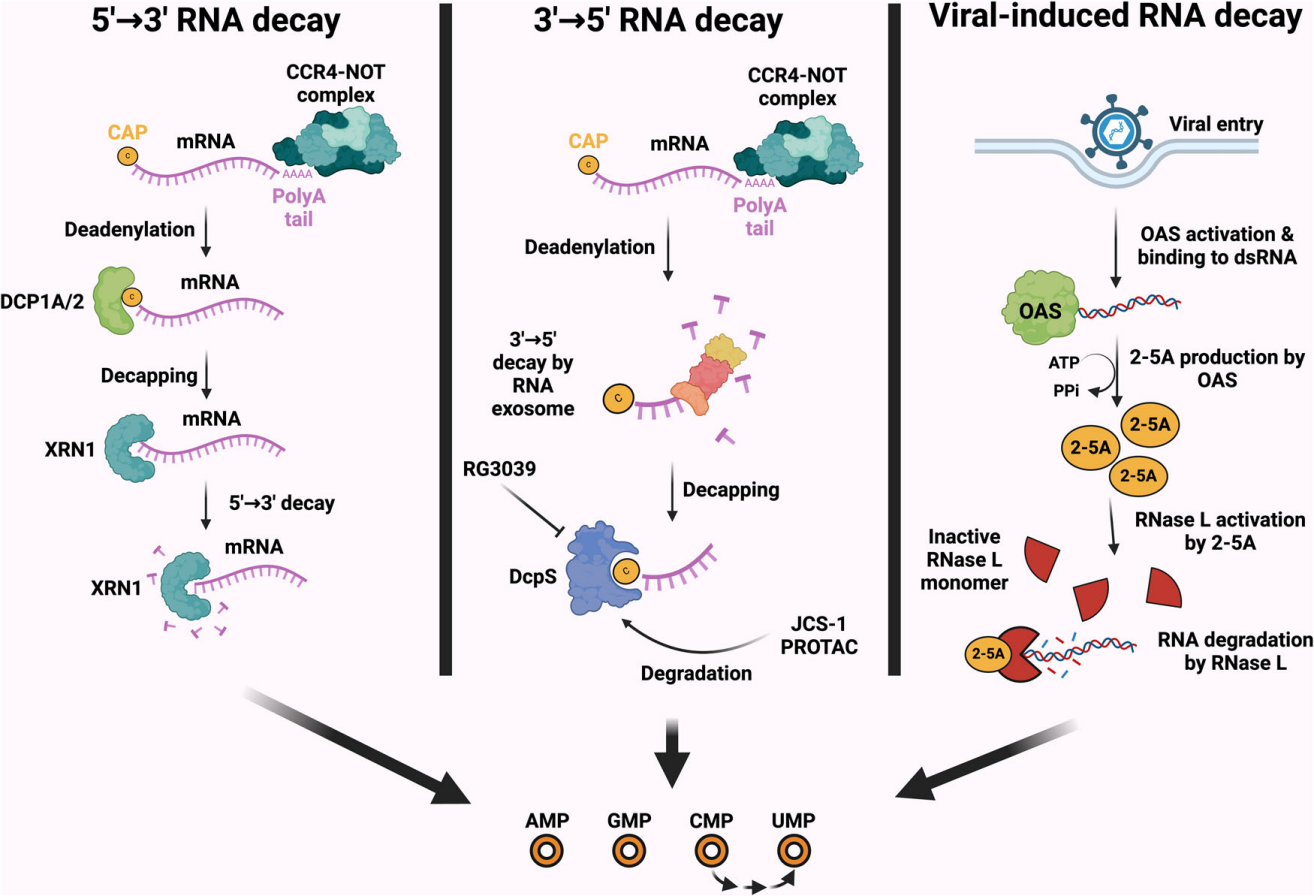
Multiple forms of RNA decay provide an intracellular source of uridine. The 3′-to-5′ decay is initiated by removal of the poly(A) tail by the CNOT complex, then decay can occur in RNA exosomes and the remaining 5′ cap is processed by DcpS. The 5′-to-3′ decay involves an initial decapping by the DCP1A/2 complex and then processing by the exonuclease XRN1. Viral-induced RNA decay involves the degradation of double-strand viral RNA by OAS enzymes, which results in 2′-5′ oligoadenylate formation, which is in turn further degraded by RNase L. In all of these cases, nucleoside monophosphates are formed, through which UMP can be directly converted to uridine. CMP can also be metabolized to UMP, providing a further source of uridine. AMP, adenosine monophosphate; CMP, cytidine monophosphate; GMP, guanosine monophosphate. Figure created using Biorender (https://biorender.com/).

In eukaryotes, most messenger RNAs are protected from degradation by a 5′-7-monomethyl guanosine (m^7^G) cap and a 3′-poly(A) tail^77–79^. RNAs are also reported to undergo distinct noncanonical 5′-end modifications, including nicotinamide adenine dinucleotide and dephospho-CoA^80,81^. RNA decay is initiated by the removal of the 3′-poly(A) tail, and deadenylated RNA can be degraded through two pathways: 3′-to-5′ RNA decay and 5′-to-3′ RNA decay^82,83^. RNA is a highly abundant molecule in cells, constituting 4–20% of dry mass^84^. mRNAs turn over faster than other types of RNA^85^, and their decay and subsequent generation of UMP may be a substantial contributor to uridine metabolism^86^. In addition, given that ribosomal RNA is the most abundant type of RNA, ribophagy resulting in rRNA degradation in vacuoles is a major source of nucleotides during nutritional stress^87^. In support of the notion of RNA as a major source, uridine production derived from RNA is efficient enough to allow the growth of cancer cells in the complete absence of glucose^24^. In the following section, we discuss what is known about the role of RNA decay processes in cancer (Fig. 5). These previous studies have elucidated how various cellular processes are modulated by these RNA decay pathways in cancer and implicated various parts of the decay machinery as potential cancer targets. We propose that these targets may have additional relevance to cancer metabolism in light of RNA being a rich source of uridine, a concept that deserves further exploration.

### The 3′-to-5′ RNA decay

Shortening and removal of the 3′-poly(A) tail of mRNA are the first steps of RNA decay, and this deadenylation is catalyzed by the deadenylase complexes PAN2–PAN3 and CCR4–NOT (CNOT)^88^. PAN2–PAN3 shortens the initial 3′ poly(A) tail, and CNOT completes the remainder of the deadenylation^89^. The CNOT complex consists of nine core subunits CNOT1, CNOT2, CNOT3, CNOT6, CNOT6L, CNOT7, CNOT8, CNOT9/RQCD1 and CNOT10 (ref. ^90^). Various CNOT subunits have been implicated in different cancer contexts. CNOT2 is implicated in breast cancer and colorectal cancer cell viability^91,92^, and its inhibition sensitizes non-small-cell lung cancer cells to TNF-related apoptosis^93^. Similarly, CNOT3 has been implicated as a driver of leukemogenesis by promoting translation efficiency^94^ and as a mediator of chemoresistance in lung cancer^95,96^. CNOT7 is involved in breast cancer metastasis^97^. The modulation of various signaling pathways, including the mTOR, STAT3, VEGF and c-Myc pathways, is associated with these effects.

Following deadenylation, mRNA can be degraded in the 3′-to-5′ direction by cytoplasmic multisubunit RNA exosome complexes^98^. The 5′ cap on the remaining oligomer is hydrolyzed by the decapping scavenger enzyme (DcpS), which converts the 5′ cap into 7-methylguanosine monophosphate (m^7^GMP) and nucleoside diphosphate^98–102^. DcpS has been shown to play an important role in mRNA homeostasis and to protect cells from the potential toxic accumulation of capped mRNA fragments^102^. DcpS enzymes, which form homodimers with two active sites situated in the grooves between the N- and C-terminal domains, can bind two cap ligands^103,104^. DcpS has recently been identified as an essential gene for acute myeloid leukemia (AML) cell survival through genome-wide CRISPR–Cas9 knockout screening. Depletion of DcpS by RG3039 or short hairpin RNA imparts potent antileukemic activity in human AML cells^105,106^. In addition, JCS-1, an E3 ligase VHL-recruiting PROTAC, has been developed to promote the degradation of DcpS, which inhibits the proliferation and viability of MOLM-14 cells^107^. These studies indicate that targeting DcpS could be an effective strategy for treating AML- and DcpS-dependent diseases.

### The 5′-to-3′ RNA decay

Unlike 3′-to-5′ decay, Dcp2 removes the m^7^G mRNA cap in 5′-to-3′ decay^108–111^, and decapped RNA is rapidly degraded by the 5′-to-3′ exonuclease XRN1 (refs. ^82,112,113^). XRN1 has recently been implicated as a cancer target whose disruption potentiates the effect of cancer immunotherapy via a mitochondrial antiviral-signaling protein-dependent mechanism^114,115^. The metabolic implications of targeting XRN1 should be explored in relation to immunotherapy, considering that targeting nucleotide metabolism could enhance cancer immunotherapy^116^.

### Endoribonuclease-mediated RNA decay

The 2′,5′-oligoadenylate (2–5A) synthetase (OAS)-–ribonuclease L (RNase L) pathway is a key component of antiviral innate immunity, which induces the degradation of viral and cellular RNAs, thereby blocking viral infection^117^. The antiviral 2–5A pathway is initiated by the binding of viral double-stranded RNA (dsRNAs) to 2′-5′-oligoadenylate synthetases, and activated OASs produce 2′-5′-linked oligomers called 2–5A [*px*5′A(2′*p*5′A)*n*; *x* = 1 − 3; *n* ≥ 2], with ATP used as a substrate^118–120^. The human OAS gene family consists of three functional genes, OAS1, OAS2 and OAS3, as well as the OAS-like gene, which encodes a protein that does not synthesize 2–5A^121^. The transcription of OAS is induced by interferon (IFN) signaling, and elevated levels of OAS contribute to the IFN-induced antiviral state^117,122^. Notably, compared with normal prostate epithelial cells, prostate cancer cells (PC3, LNCaP and DU145 cells) overexpress mRNAs encoding proteins that bind and activate OAS, such as Raf kinase inhibitor protein (RKIP), poly(rC)-binding protein 2 (PCBP2) and human endogenous retrovirus (hERV) envelope RNAs^123,124^. This finding indicates that OAS could be activated independently of virus infection in cancer.

RNase L, known as an IFN-induced endoribonuclease, is activated by 2–5A produced by OAS, resulting in the cleavage of all RNAs in the cell^123,125^. RNase L suppresses the mobile genetic element LINE-1 and stimulates apoptosis, inflammation and autophagy^123,126–129^. RNase L knockdown by shRNA increases the migration of both human prostate cancer cells and mouse embryonic fibroblasts^123^. In addition, the overexpression of RNase L inhibits androgen receptor signaling and migration in prostate cancer cells^130^. Considering these findings, the mutation status of the RNase L gene might be a key factor in promoting cell migration and metastasis in prostate cancer. Notably, IFN-γ enhances the expression of RNase L and restores its function in the cytoplasm and nucleus, which leads to apoptosis in lung cancer cells^131^. These findings indicate that RNase L could act as an adjuvant to enhance the efficacy of cancer immunotherapy via IFNs such as IFN-γ.

**Fig. 6:**
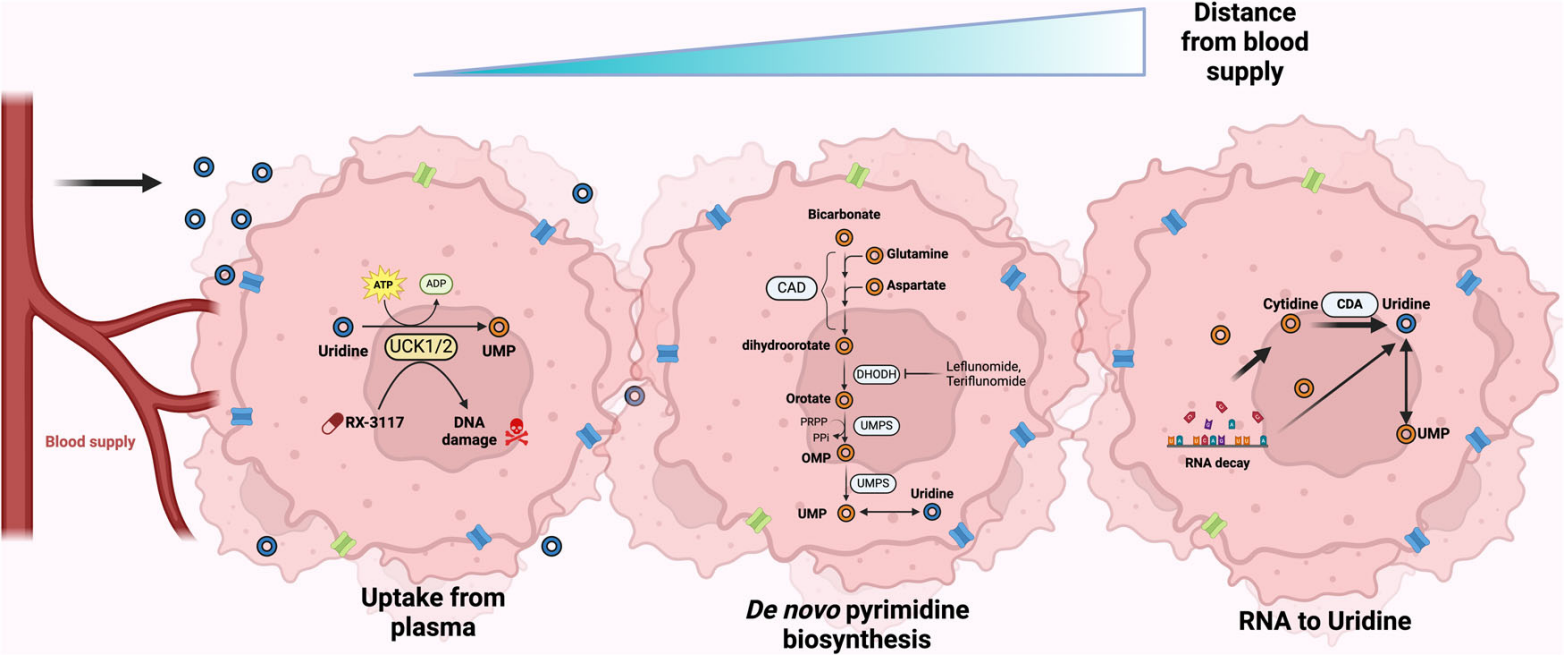
Predicted model for uridine uptake mechanism according to distance from blood supply. The upstream sources of uridine may depend on the availability of uridine itself or various precursors and, in states of depletion, RNA decay may provide a key source of uridine. Figure created using Biorender (https://biorender.com/).

On the basis of these various potential sources of uridine for cells, we propose the following model for how cancer cells within solid tumors obtain uridine (Fig. 6). In tumor cells that are proximal to working vasculature, uridine should be readily obtained from the blood supply (Fig. 6, left). They may additionally obtain uridine from the pyrimidine biosynthesis pathway, which is upregulated in proliferating cells to meet the nucleic acid demands of a growing cell^132^. However, in a poorly vascularized tumor or a cancer cell that is distal to the blood supply, it is likely that not only glucose but also precursors (for example, aspartate and glutamine) for pyrimidine biosynthesis are highly limited. Therefore, recycling from the abundant RNA molecules in the cell is probably a critical source of uridine and downstream products in these cells. This model is suggested based on recent studies showing that exogenously provided uridine or RNAs can provide an alternative fuel source under glucose limitation^24,25^. However, further studies are needed to directly examine the importance of RNA decay in providing uridine as an alternative fuel source in the starved tumor microenvironment.

## Part 4: downstream fates of uridine to fuel cancer cell processes

### Uridine contribution to glycolysis and the PPP

**Fig. 7:**
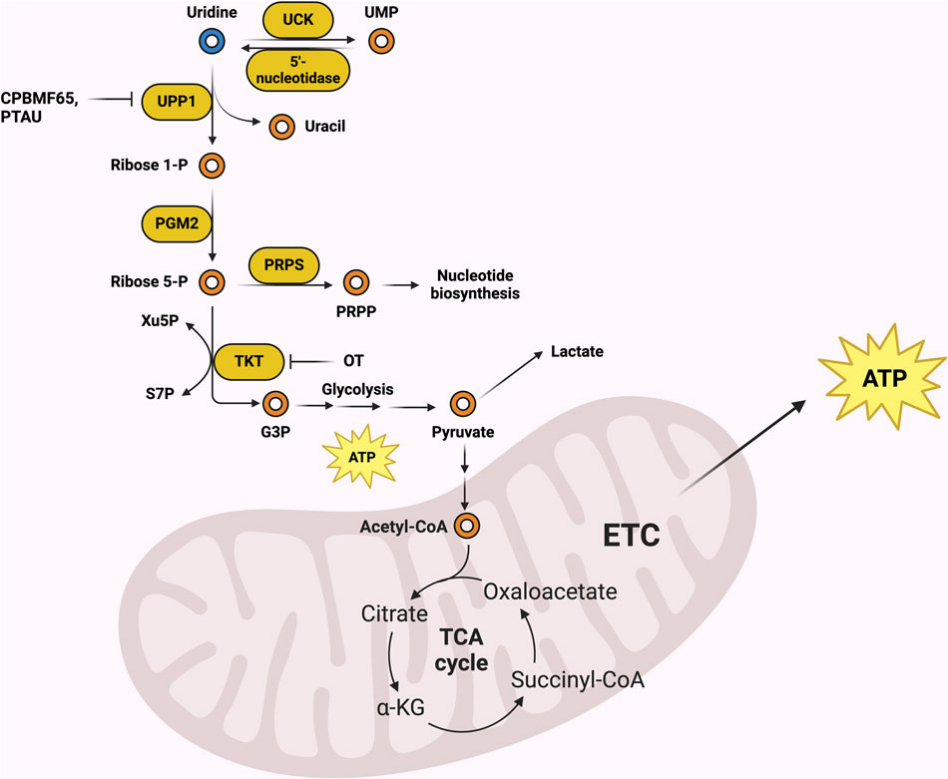
Uridine catabolism can contribute to central carbon metabolism and the TCA cycle. The pathways and drugs that can target specific steps are outlined. Figure created using Biorender (https://biorender.com/).

Recently, uridine was shown to be a key contributor to central carbon metabolism, particularly when glucose is limited for tumor cells. Uridine can be converted into ribose 1-phosphate and uracil by uridine phosphorylase (UPP)^25^, and it can be phosphorylated to produce UMP via UCK^49,50,133^. The removal of the phosphoryl group from UMP by 5′-nucleotidase results in uridine, and the conversion of UMP to OMP is mediated by orotidine 5′-phosphate decarboxylase in de novo synthesis^134^. Recently, it has been reported that ribose derived from uridine by UPP1 can overcome the growth defects induced by glucose deficiency in pancreatic cancer and that high UPP1 expression predicts poor outcomes in patients with pancreatic cancer^24,25^. Thus, uridine metabolism can be considered a source of fuel for cancer proliferation, emphasizing its potential as an anticancer target (Fig. 7).

### Uridine to uracil and ribose 1-phosphate by UPP

Glucose, a primary carbon and energy source for cell growth, proliferation and activity, is metabolized through multiple pathways, including glycolysis and the pentose phosphate pathway (PPP)^135^. The increased uptake and dependency of cancer cells on glucose and their tendency to secrete carbon as lactate even under aerobic conditions were key early observations in the cancer metabolism field^136^. These properties are exploited in tumor imaging via ^18^F-fludeoxyglucose–positron emission tomography^137^. Blocking the uptake or metabolism of glucose is considered a strategy for inhibiting tumor growth^138–140^. However, an insufficient glucose supply is a situation commonly encountered by solid tumors, and cancer cells can utilize nutrients such as amino acids, lactate, acetate and other macromolecules as alternative fuels^141^.

Recently, uridine has been shown to serve as an alternative fuel for cancer growth in the absence of glucose^24,25^. In this process, uridine is converted to uracil and ribose 1-phosphate by UPP. In vertebrates, there are two forms of the phosphorylase, UPP1 and UPP2 (ref. ^142^). UPP expression and activity are induced by redox or chemotherapeutics; UPP2 activity is affected by the redox state due to a disulfide bridge, and the KRAS–MAPK pathway and NRF2 can modulate UPP1 expression^25,142,143^. The steady-state level of *UPP1* mRNA is positively correlated with the poor prognosis of patients with multiple types of malignant tumors, including breast cancer, lung adenocarcinoma and oral squamous cell carcinoma^144–146^. Owing to the role of UPP1 in uridine usage under glucose-limiting conditions, how UPP1 is regulated and its implications for tumor outcome remain important topics for continued investigations.

Targeting UPP enzymes has previously been shown to impair various functions of cancer cells, which may in part include the interaction of UPPs with other proteins. UPP1 knockdown by siRNA significantly suppresses the migration, invasion and proliferation of TPC and BCPAP thyroid carcinoma cells^147^. Similarly, the pharmacological inhibition of UPP1 using CPBMF65, a synthetic human uridine phosphorylase-1 inhibitor, reduces the proliferation of HepG2 cells, leading to cell cycle arrest and senescence^148^. In lung adenocarcinoma, UPP1 knockdown via shRNA reduced the protein levels of ENO1 and LDHA in H292 and H1975 cells, whereas overexpression of UPP1 restored the suppression of lactate production, glucose uptake and ENO1/LDHA protein levels induced by 2-deoxy-d-glucose in H1299 cells^145^. A specific inhibitor of UPP, 5-(phenylthio)acyclouridine (PTAU), attenuated the reduction in glucose deprivation-mediated cell death caused by uridine in lipopolysaccharide- and IFN-γ-treated astrocytes^149^. In addition, UPP1 binds the C-terminus of AKT and increases the activity of AKT by promoting the interaction of AKT with PDK1 and PDK2 and recruiting phosphatidylinositol 3,4,5-triphosphate to AKT^150^. This is related to tumorigenesis and resistance to the drug gemcitabine in bladder cancer^150^. UPP2 has the potential to sense and initiate cellular responses to oxidative stress^142^. In addition, hepatic nuclear receptor agonists have been reported to regulate mouse UPP2, implying an additional function of UPP2 in hepatic lipogenesis or cholesterol transport^142,151,152^. In summary, multiple metabolic and signaling mechanisms justify UPP1 as an attractive target for anticancer treatment strategies.

### Ribose 1-phosphate to R5P by PGM2

Phosphoglucomutase (PGM) catalyzes the reversible conversion between glucose 6-phosphate and glucose 1-phosphate using Mg^2+^ (refs. ^153–155^). The PGM protein family members PGM1–5 share homologous coding sequences, but their substrates and functions vary^154^. In the presence of uridine and the absence of glucose, phosphoglucomutase 2 (PGM2) connects the UPP1/UPP2 reaction to the PPP by catalyzing the conversion of ribose 1-phosphate to ribose 5-phosphate (R5P)^24^. Skinner et al. reported that PGM2 is essential for survival and proliferation in glucose-deprived, uridine-rich media, but is dispensable in glucose-containing media, indicating its essential role in the utilization of uridine as an energy source in the absence of glucose^24^. Additionally, *PGM2* mRNA accumulates in cells of various cancers (lung, central nervous system, pancreas and small/large intestine), and this is associated with an unfavorable prognosis in patients with lung adenocarcinoma^156^. Considering these findings, targeting PGM2 could effectively inhibit uridine metabolism and be utilized as a target in treating cancer.

### R5P to glyceraldehyde 3-P by TKT

Transketolase (TKT), a key enzyme of the nonoxidative PPP, catalyzes the transfer of two carbons from xylulose 5-phosphate to the co-factor of TKT, thiamine pyrophosphate, which then delivers them to R5P or erythrose 4-phosphate. R5P and erythrose 4-phosphate are converted into sedoheptulose 7-phosphate (S7P) and fructose 6-phosphate, respectively, and xylulose 5-phosphate is converted into glyceraldehyde 3-phosphate^157,158^. Glyceraldehyde 3-phosphate is subsequently converted into pyruvate through the second phase of glycolysis, which is then converted into acetyl-CoA, allowing it to enter the TCA cycle for ATP production^159,160^. TKT is transcriptionally regulated by various biomolecules, including HIF-1α, miR-497 and steroid receptor coactivator-3 (refs. ^157,161–165^). The transcription of TKT in imatinib-resistant chronic myelogenous leukemia cells is regulated by HIF-1α, sustaining the survival and proliferation of these resistant cells^166^. Transfection of miR-497 mimics targeting *TKT* mRNA increases the sensitivity of HeLa and SiHa cells to cisplatin, increasing their viability^164^. PFKFB4 promotes TKT transcription by activating oncogenic steroid receptor coactivator-3, which enhances breast tumor growth and metastasis in immunocompromised mice^165^.

Furthermore, TKT is overexpressed in various cancers, including colorectal, pancreatic, breast and lung cancer, and patients with elevated *TKT* mRNA levels have decreased survival rates compared with those with lower levels^157^. The representative TKT inhibitor oxythiamine (OT) has been shown to inhibit tumor proliferation by blocking the transition of thiamine to its active form, which is required for the full activity of TKT^157,165,167,168^. Like PGM2, TKT was found to be important in uridine utilization as an energy source in the absence of glucose^24^. These results indicate the critical function of TKT in uridine utilization in glucose-deficient cancer cells and suggest its potential as an anticancer target.

### R5P to PRPP by PRPS

PRPP synthetase (PRPS) converts R5P into PRPP, which is utilized in nucleotide synthesis^169–171^. In pyrimidine synthesis, the pyrimidine ring is first synthesized and then added to PRPP^170^. Purine synthesis occurs entirely in the cytosol and the purine ring is directly built onto PRPP^172^. Hyperactive nucleotide synthesis in cancer cells is a common characteristic, and cancer cells have higher concentrations of dNTPs and NTPs than nonmalignant proliferating cells do^2,71^. These findings could lead to the development of anticancer agents that target the enzymes involved in nucleotide synthesis. Notably, the loss of TKT has been reported to suppress glycolysis and the TCA cycle, resulting in a reduction in ATP levels and the inhibition of PRPS^173^. These findings indicate that targeting TKT could lead to the inhibition of PRPS and nucleotide synthesis.

### Uridine contribution to glycosylation and phase II detoxification

Another important facet of uridine may be its role in glycosylation, in which sugar nucleotide molecules are utilized as sugar donors. There are 12 sugar nucleotides used in glycosylation reactions in humans, seven of which are UDP bound, including UDP–glucose (UDP–Glc), UDP–glucuronic acid (UDP–GA) and UDP–*N*-acetylglucosamine (GlcNAc)^174^. The formation of UDP sugars and subsequent glycosylations are thus tied to uridine metabolism to form UMP and ultimately uridine triphosphate (UTP), which is then used in the UDP sugar-forming condensation reactions. For example, UDP–glucose pyrophosphorylase (UGP) interconverts UTP and glucose 1-phosphate to form inorganic pyrophosphate (PPi) and UDP–Glc^175^. UDP–Glc can further be converted to UDP–GA via UDP–glucose 6-dehydrogenase (UGDH)^176,177^.

Thus, uridine formation or uptake is important for the formation of the UDP sugars required for glycosylation. Studies of in vitro cell models have demonstrated that the addition of uridine to cell culture results in dramatic increases in both UDP and cytidine diphosphate sugars and their corresponding glycosylations^178,179^. Indeed, systemic uridine administration results in an increase in UDP–Glc and UDP–GlcNAc, as well as GlcNAc-glycosylated proteins^36,54^. Moreover, mitogen stimulation of quiescent cells results in rapid uptake of uridine, which is incorporated into UDP–GA^180^. These studies indicate that uridine uptake and/or production can be rate limiting in the formation of UDP sugars and the subsequent glycosylation reactions in in vitro tissue culture and in vivo mouse models. Thus, disrupting the contribution of uridine to UDP sugars may have widespread implications for cancer cells, as the glycosylation of proteins and other biomolecules impacts all aspects of the cancer cell surface, including attachment, invasion, surface receptor activity and even interactions with immune cells^174^. For example, elevated GlcNAc glycosylation of PD-L1 is utilized by cancer cells in immune evasion^181^; thus, uridine metabolism may contribute to such processes.

Furthermore, the proper glycosylation of proteins is critical for preventing their misfolding and misprocessing; thus, any disruption of the process may induce or sensitize cancer cells to stress on the endoplasmic reticulum (ER) or dysfunction of the Golgi, key sites of glycosylation. In support of the relationship between uridine and ER stress, the unfolded protein response can trigger uridine biosynthesis, whereas disruptions in the uridine–UDP sugar axis can trigger ER stress or Golgi dysfunction^177,182,183^.

Among the UDP sugars impacted by uridine metabolism, UDP–GA may be particularly relevant to cancer therapy. The formation of UDP–GA is increased in cancer cells^177,184^, which may be beneficial for tumors via multiple mechanisms, including extracellular matrix modulation, epithelial‒mesenchymal transition and chemoresistance^177,185,186^. UDP–GA is processed into UDP–xylose by the enzyme UDP–xylose synthase (UXS1), and when UXS1 is disrupted, UDP–GA selectively accumulates in such cancer cells, poisoning them and disrupting their Golgi structure and function^177^. The notion that excess UDP sugars exert toxic effects suggests that excess production of toxic byproducts may be relevant to the toxicity observed in the presence of excess uridine^10,11^ and that this could be exploited to exert toxicity in cancer cells.

Uridine metabolism may also be linked to chemoresistance by virtue of its role in UDP–GA production. UDP–GA is the substrate used in a process called ‘glucuronidation’, which is carried out by UDP–glucuronyltransferases (UGTs), a family of 22 enzymes that constitute a phase II detoxification mechanism. The enzymes have differing substrate specificities and can glucuronidate some normal metabolites in addition to xenobiotics (for example, bilirubin)^187^. In contrast to phase I detoxification (modification), which occurs primarily in the liver, phase II detoxification activity is spread across multiple organs and in cancer cells themselves^188^. In this reaction, glucuronic acid is conjugated by UGTs to xenobiotic metabolites, which are selectively recognized by the different UGTs. This conjugation has the effects of neutralizing reactive electrophiles and thus detoxifying the compound, as well as rendering them polar and conducive to transport outside of the cell. As such, phase II detoxification represents a mechanism that can increase the chemoresistance of cancer cells. Indeed, various chemotherapeutics have been shown to be glucuronidated and various individual UGTs have been shown to be upregulated in specific tumors. For example, UGT1 and UGT2 isoforms, such as UGT1A1, UGT1A6 and UGT2B17, have been shown to be overexpressed or induced by chemotherapeutics in tumors and can reduce the activity/levels of various chemotherapeutic agents, including irinotecan, sorafenib, raloxifene and tamoxifen^188^. Additionally, targeting individual UGTs has been shown to induce chemosensitivity in cancer cells^188,189^.

**Fig. 8:**
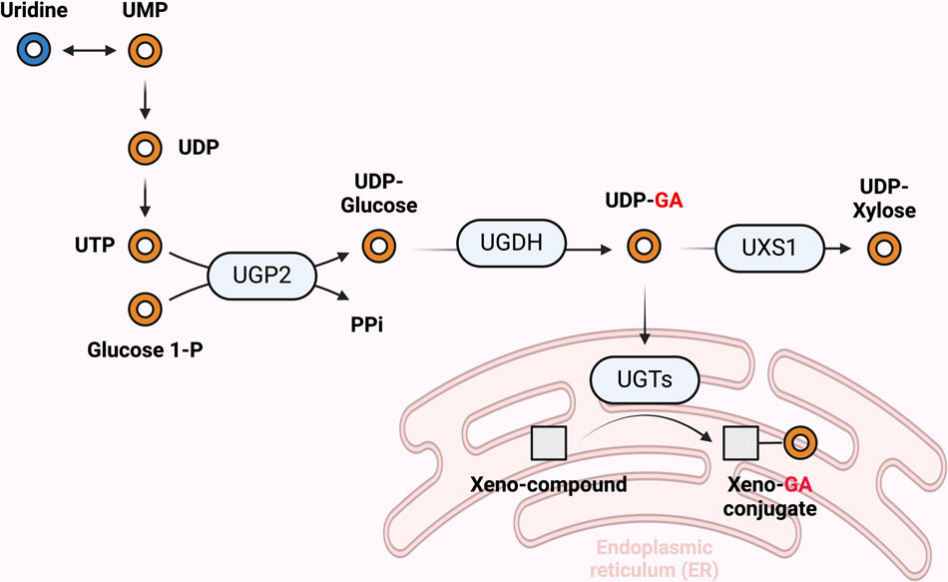
Pathway for uridine contribution to a subset of UDP sugars and glucuronidation reactions. Uridine is ultimately metabolized to UTP to form UDP–glucose, which in turn can be converted to other sugars, including the glucuronidation substrate UDP–GA. Figure created using Biorender (https://biorender.com/).

Uridine has long been indicated as a ‘rescue agent’ against nucleoside chemotherapeutics because of its competitive effect on incorporation into RNA^40^. The link between uridine metabolism and the sugar nucleotide UDP–GA opens a new line of thinking and direction for future research into how uridine metabolism may regulate the chemoresistance of cancer cells via UDP–GA and glucuronidation; thus, targeting uridine or the various enzymatic steps between uridine and UDP–GA formation could be explored as chemosensitization approaches (Fig. 8).

## Conclusions

Uridine is not only an organism-level ‘currency metabolite’ that is readily available to cells via the plasma^4,190^, but also a ‘hub molecule’ inside the cell that intersects with numerous intracellular processes^24,36,178,179^. Uridine can further be obtained via the breakdown/salvage of RNA^108,123,191^ or via pyrimidine biosynthesis^69,192^, both of which are highly active processes in cancer cells^69^. Conversely, uridine itself is a necessary precursor to many biological processes that are central to cancer cell biology. Uridine-derived ribose enables cancer cells to generate energy under conditions of glucose deficiency, contributing to their survival and proliferation^24,25^. Uridine is rate limiting in the formation of sugar nucleotides that are central to glycosylation reactions^54,178,180^ and may even be directly linked to chemoresistance because of its metabolic role in the formation of the key phase II detoxification molecule UDP–GA^177,186,188^. The various enzymes involved in RNA breakdown/salvage, or pyrimidine biosynthesis in many cases, have long been explored for various reasons as cancer therapeutics^71,75^, but their roles specific to uridine metabolism have not previously been investigated and represent new opportunities to better understand the cancer-related contexts in which they may be most useful. In particular, the recently demonstrated role of RNA polymers as cellular sources of uridine^24^ indicates that further research is needed into RNA metabolism processes as upstream precursors to uridine metabolism. The enzymes that mediate the processes downstream of uridine leading to glycolysis or UDP sugars^24,25,177^ have only recently been identified and are also excellent targets for disrupting processes that are integral to cancer cell function.
